# Descriptive Account of a Bicephalous Fœtus

**Published:** 1821-02

**Authors:** I. Jackson

**Affiliations:** Member of the Royal College of Surgeons of London.


### *Descriptive Account of a Bicephalous Fœtus.* By Mr. I. Jackson, Member of the Royal College of Surgeons of London.

MARY HONEYFORD, the mother of the child to be described, is unmarried, about twenty years of age, of a sanguineous temperament, florid complexion, and rather below the middle stature; her occupation is that of a weaver; and she has enjoyed nearly uninterrupted good health from infancy.

In the forenoon of the 10th of August, 1820, I was desired to visit her, and take with me the midwifery instruments. I learnt from the messenger, that she had been in labour upwards of two days, and that midwives had been with her most of the time. For the last eighteen hours, one of them had been constantly assisting her, but was unable to accomplish her delivery: the pains were very weak, and her strength nearly exhausted.

When I arrived at the house, I was informed by the midwife that the waters had been drained off twenty or twenty-four hours; the head had been in the situation it now occupied for ten or twelve hours; and, to use her own expression, the head had descended slowly to the birth, and then it had stuck on the spare bone.

The patient's pulse was 135, and weak. The parturient paroxysms occurred every three minutes, but were inefficient. Anxiety was depicted in her countenance, and a general restlessness pervaded her; as is frequently, if not always, witnessed in tedious painful labours.

On examination *per vaginam*, I found the os frontis presenting, the integuments of which were tumefied, from the time it had remained in the pelvis, and the attempts the midwife had made to assist her. The face was towards the pubis, or rather towards the right groin, and the soft parts of the mother quite relaxed: thus, reasoning from my experience in similar presentations, I believed there could be no apparent obstacle to a speedy and favourable termination. I ordered her a little wine and water, and calmed her mind, by flattering her with a speedy release from her suffering and anxious situation.

As the parturient energies were nearly exhausted, I was persuaded it was not safe to trust any longer to nature, and that artificial aid was necessary to assist the natural efforts to promote her delivery: I therefore assisted her in the following manner. I introduced two fingers under the os pubis of the mother, where I could reach the mouth of the child, by which I could command considerable force, and assist the expulsive efforts of the uterus: thus, by extracting at each pain, in less than half an hour the face emerged from under the pubis, and the head was nearly half protruded through the os externum. Finding that something impeded the complete expulsion of the head, though I used considerable extracting force, I passed two fingers by the head of the child to ascertain the obstruction, and where it existed, when they came in contact with a firm tumor, descending into the cavity of the pelvis, closely joined in contact with the neck of the first head. On examining it as particularly as the confined state of the parts would permit, I was convinced it was the head of a child, from the hair on the scalp and the feel of a suture.

I did not recollect the record of any case similar; for I imagined it a case of twins, in which the head of the second child, by some partial contraction of the uterus, had been forced down, or somehow got entangled before the shoulder of the first. After considering for a short time what would be the most preferable method to pursue to accomplish a safe delivery, I came to this determination, that, as the superior aperture of the pelvis was sufficiently capacious to admit the head and neck of a child in conjunction, I would attempt to extract the child whose head was already in part protruded; and, if I failed in that attempt, I would perforate the second head in the pelvis, and break it down with the blunt hook, as the most probable means of safety to the mother.

The woman continued to have regular, though very inefficient, pains, at each of which I used such extracting force as I thought was compatible with the safety of the mother and child: I easily ascertained it was living, by the pulsation of the temporal arteries. After some time I found I was gaining a little ground, and that the heads retained their relative situation; that is, as one was further protruded, the other descended lower towards the os externum. Having more space for examining the presenting part of the second head, I satisfied myself, from the sutures and posterior fontanell, that it was the occiput.

I thus continued extracting and guarding the perineum for about an hour, when the second head was protruded; immediately after which followed a sharp expulsive pain, when the body of a living male child with two heads was brought to view.

The child appeared lively. I lost no time in tying the umbilical cord, which was no thicker than ordinary, that I might remove it, to examine it more particularly and make further observations.

Having taken it into an adjoining room, I found it had all the powers of voluntary motion as perfect as a natural fetus: the eyes of each head were opened and closed occasionally; and the muscles of each face contracted, as if to squall, and one of them made a considerable noise, which was the first head protruded. On a closer inspection, I found the other head never breathed, although it was equally lively with the first. It continued gradually to weaken for about forty minutes, when it ceased to breathe. For a considerable time after respiration had ceased, I could feel the heart palpitating, with a tremulous motion, in the epigastric region.

With respect to the mother, I may briefly state, that, whilst I was making the above remarks on the child, one of the midwives, by officiously attempting to bring away the placenta, broke the umbilical cord; so that I was obliged to introduce my hand into the uterus, to detach and bring it away. Notwithstanding this circumstance, added to the tedious and painful parturition, she recovered as well as after an ordinary labour: no unpleasant symptoms supervened, except that the prostration of strength required a longer time to be re-established.

Mr. Bailey, of Blackburn, a respectable surgeon and good anatomist, assisted me in examining the child, about twenty-four hours after its birth; when we made the following observations.

*External appearances.*—The heads were well formed; the bones of which were as perfectly ossified, as well as those of the limbs, as is found in the majority of infants. The neck of the left head appeared somewhat longer than the other; but I attributed that to the extending force I used in the extraction; and the other would be forced in an opposite direction upon the breast, by the resistance given from the soft parts of the mother. It had clavus of the right foot. The weight of the child was eight pounds, fifteen ounces, and six drachms, avoirdupoise.

The measurement of the left and larger head, from the sinus frontalis to the tubercle on the os occipitis, 8½ inches; round the head, 13 inches, at the same relative points. The smaller head measuring 7¼ inches, and 12½ inches. The circumference of the chest immediately below the arms, 14 inches. The sternum was broader than is usual, and the ribs appeared to make a greater curve than is generally observed. The claviculæ were three; two in their natural situations; and the third, which was equally as large as the others, was attached to the top of the sternum, and proceeded backwards between the two necks; where there was also a third scapula, forming a protuberance, or shoulder.

On examining the back, there felt as if there were three vertebral columns: the middle one, on dissection, was found to be the cartilages of ribs, each about an inch in length, coming from the two spines, which joined at obtuse angles, and gave to the touch externally the feel of spinous processes.

*Internal examination.*—On opening the thorax, the first thing that attracted our attention was the situation of the heart in its pericardium: it was situated between the two lungs, and about as much inclined to the right side as in a natural case it is to the left. The lungs were large, having two tracheæ, each terminating separately; the one in the right, the other in the left, lung. Each lung had three lobes. Each head had a distinct œsophagus, which passed separately through the diaphragm. The appearance of the lungs confirmed the observation I before made, that only the left head had breathed; the right lung having the appearance of liver, the colour of which, on being inflated, was instantly changed to that peculiar pulmonic mottled hue, which characterizes this organ after respiration.

On opening the pericardium, the heart was found to be very large. The aorta, which was also very large, arose from the left ventricle; as also did the pulmonary artery to the left lung. The aorta ascended about an inch, then made a turn backwards by the left side of the trachea at the root of the right spine, towards the right side, receiving the sinus arteriosus, from the left pulmonary artery, and giving off an artery which ramified on the short ribs connecting the vertebræ. It then passes to the right spine, on the left side of the œsophagus, and forms behind that organ a beautiful arch; and, with another artery, arising from the right ventricle, and which I will call a minor aorta, forms a large common aorta descendens.

The right ventricle gives origin to the right pulmonary artery, and also to another which may justly be called a minor aorta: the latter ascends about one inch, passing from left to right in front of the right trachea, giving off an artery which subdivides into two,—a small subclavian to the third clavicle, and the left carotid of the right head. Having passed across the trachea, it gives off an arteria innominata, which bifurcates into the right subclavian and right carotid of the right head. The continuation of the aorta then passes to the spine, on the right side of the trachea, receiving a sinus arteriosus from the right pulmonary artery, and joins the large aorta from the left ventricle, forming together, as before observed, the aorta descendens.

The aorta descendens then passes down on the left side of the right spine as low as the first lumbar vertebræ, when it gives off a large artery, which may very properly be denominated an aorta ascendens. Passing up as high as the first cervical vertebræ on the left side of the spine belonging to the left head, it then subdivides into two common trunks; the left of which immediately bifurcates with the left subclavian and carotid. The right trunk ascends about an inch under the trachea and œsophagus, and then divides into the right carotid of the left head, and a subclavian to the common clavicle. Thus the third clavicle has two subclavian arteries, but smaller than those which supply the extremities, and which inosculate in a beautiful manner.

The aorta descendens, after having given off the aorta ascendens, descends upon the spine, giving off the usual abdominal arteries, and bifurcates, as is natural, into the two common iliacs.

In the venous system, nothing particular or extraordinary was observed; the jugular and subclavian veins of each head and extremity united to form one great vena cava superior. The vena cava inferior was single, and of the usual magnitude.

The contents of the abdomen appeared natural: the liver was large, but had nothing peculiar in its appearance. On turning back the liver, we found two stomachs: the one to the left head occupied the natural situation, the other was situated under the right lobe of the liver. Each stomach had its separate duodenum, the right one about half an inch, and the left one about an inch and half in length; when they united, forming one common duodenum. There were two gall-bladders, each having a separate duct, which terminated at the junction of the duodena. The spleen, pancreas, and kidneys, were natural; as also were the intestines; the colon and rectum were distended with meconium. The contents of the pelvis were similar to those of a single child.

On examining the vertebral columns, we found two, perfect and entire, but which gradually approximated as they approached the sacrum; which was single, but much broader than natural.

Having given a detail of the circumstances attending the labour, with the impressions they made on my mind at the time of their occurrence, the observations I shall make will be very concise.

One head appears something larger than the other; but I believe this circumstance arose from the difficulty of the labour. The one, having presented at the os externum for several hours, was tumefied; whilst the other, from excessive pressure, had the sutures considerably overlapped. The difference in the length of the necks, I have observed, arose from the extension I used to effect the delivery of the one, with the counter-pressure upon the other.

The child was well grown, as its weight intimates; the members were well formed; and the arteries and nerves proportionate to the parts they supplied.

"The annals of the history of man," says M. Fournier, "are filled with extraordinary facts of the aberrations of nature from the ordinary state in the phenomena of conception."

Deviations from the strictly natural figure, to the practitioner in midwifery, are not unfrequently met with. The most common are supernumerary toes and fingers, malformation of the parts of generation, and spina bifida. Cases similar to the present very rarely occur: few so singular are on record; and, of those related, but a small share are well authenticated, or else they were expelled before the full time of utero-gestation. Yet the history of rare and uncommon cases, the truth of the major part of which cannot be doubted, with valuable and interesting remarks, are not wanting.* A case similar to the one I have related, with a few exceptions, was recorded not long ago by that late excellent anatomist, Paul Mascagni.† It ap-

* See an article, entitled *Cas Rares*, in the *Dictionnaire des Sciences Medicales*, par M. Fournier. Also, Mr. Mason Good's *Physiological Nosology*.

† *Giorn. della Soc. Medic. Chirurg. di Parma*, vol. xv.

pears, from his description, that its external conformation was exactly the same; the difference existed in the viscera and circulating system.

It was my intention to have procured this fetus, and to have presented it to the College of Surgeons; but, public curiosity being excited from such an extraordinary circumstance, the father of the woman has contrived to make it a source of gain. It is beautifully preserved in spirits, and they are now exhibiting it as a curiosity in the neighbouring towns.

*Bolton-le-Moors, Lancashire; Oct.* 1820.

